# Hypoxia and the Aging Cardiovascular System

**DOI:** 10.14336/AD.2023.0424

**Published:** 2023-12-01

**Authors:** Antoine Raberin, Johannes Burtscher, Martin Burtscher, Grégoire P. Millet

**Affiliations:** ^1^Institute of Sport Sciences, University of Lausanne, CH-1015, Lausanne, Switzerland.; ^2^Department of Sport Science, University of Innsbruck, Innsbruck, A-6020, Austria.

**Keywords:** elderly, altitude, blood pressure, hypoxia conditioning, older individuals

## Abstract

Older individuals represent a growing population, in industrialized countries, particularly those with cardiovascular diseases, which remain the leading cause of death in western societies. Aging constitutes one of the largest risks for cardiovascular diseases. On the other hand, oxygen consumption is the foundation of cardiorespiratory fitness, which in turn is linearly related to mortality, quality of life and numerous morbidities. Therefore, hypoxia is a stressor that induces beneficial or harmful adaptations, depending on the dose. While severe hypoxia can exert detrimental effects, such as high-altitude illnesses, moderate and controlled oxygen exposure can potentially be used therapeutically. It can improve numerous pathological conditions, including vascular abnormalities, and potentially slows down the progression of various age-related disorders. Hypoxia can exert beneficial effects on inflammation, oxidative stress, mitochondrial functions, and cell survival, which are all increased with age and have been discussed as main promotors of aging. This narrative review discusses specificities of the aging cardiovascular system in hypoxia. It draws upon an extensive literature search on the effects of hypoxia/altitude interventions (acute, prolonged, or intermittent exposure) on the cardiovascular system in older individuals (over 50 years old). Special attention is directed toward the use of hypoxia exposure to improve cardiovascular health in older individuals.

## 1. Introduction

Oxygen consumption (limited by both convective and diffusive factors) is the foundation for cardiorespiratory fitness, which in turn is linearly related to mortality, quality of life and numerous morbidities [1]. Beside exercise, the prolonged exposure to reduced ambient oxygen pressure (i.e., hypoxic conditions) has the potential to improve the body’s oxygen transport and utilization capacity, including via respiratory adaptations, vascular remodeling, hematological changes and improved efficiency of oxygen-dependent molecular processes [2]. While severe hypoxia can exert detrimental effects, such as acute mountain sickness (AMS), high-altitude pulmonary - or cerebral - edema (HAPE and HACE, respectively) [3], moderate and controlled oxygen exposure can potentially be used therapeutically [4, 5].

**Figure 1.**
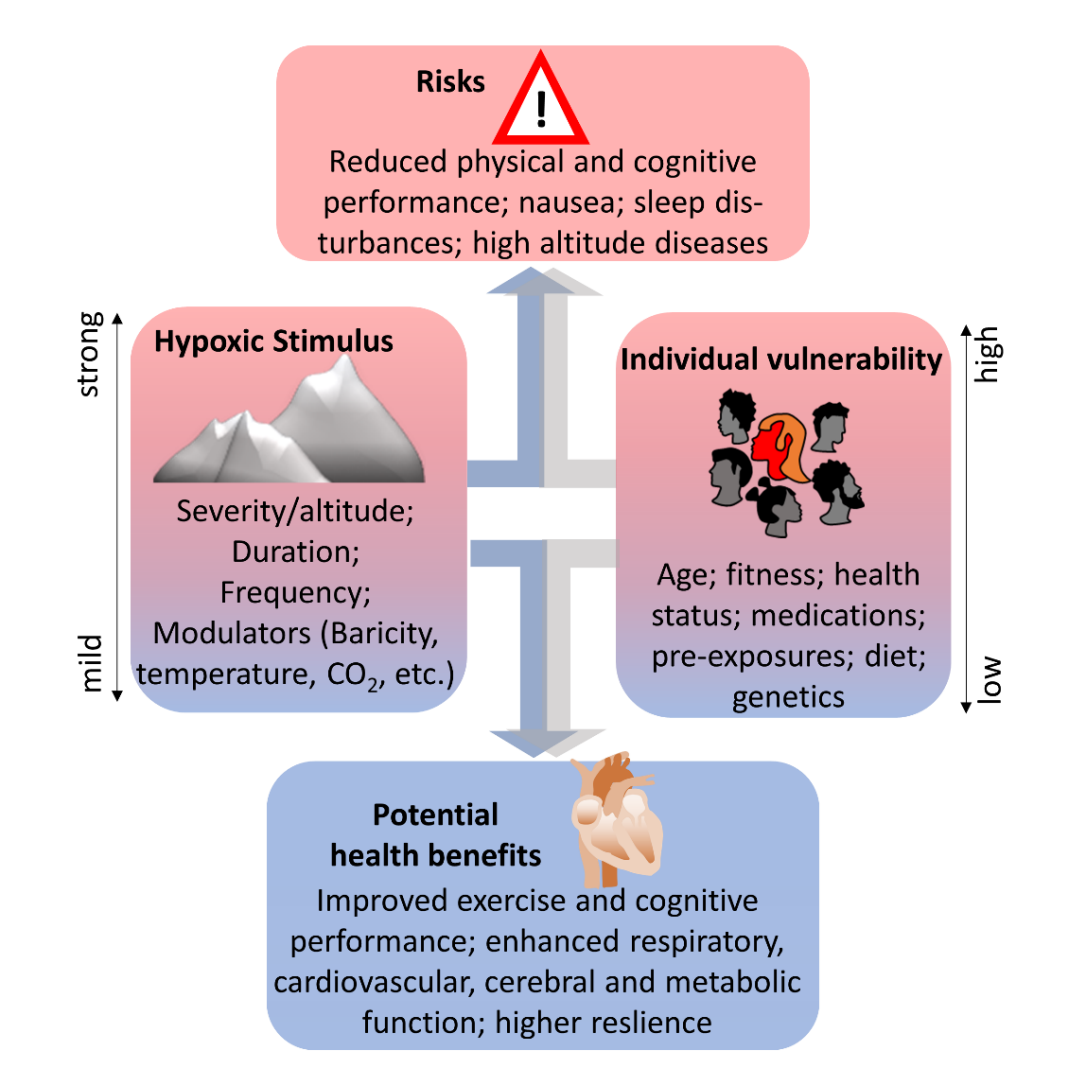
**Influences of stimulus parameters and condition of the subject (“resilience”) on the quality of the effects of hypoxia exposure**. If the stimulus is mild enough, beneficial adaptations may be induced that in turn increase resilience. If the severity of the stimulus surpasses the organism’s tolerance capacity, harmful effects might arise.

Different parameters of hypoxic exposure (including severity, duration, and frequency) dictate, whether hypoxic stimuli improve or deteriorate health [6, 7]. The hypoxic stimuli induce molecular and physiological adaptations in subjects that can prime them for later harmful insults in a process referred to as hormesis [8], as long as the stimulus is not already harmful by itself. In addition, individual resilience/vulnerability determines whether a hypoxic stimulus induces damage or triggers fortifying adaptations. One of these conditions is age (Fig. 1).

We have previously reviewed how hypoxia may be involved in the pathogenesis of various age-related blood rheological disorders [9] and diseases of the brain, one of the most oxygen-dependent organs of the human body and, conversely, how beneficial adaptations to hypoxia may be neuroprotective in elderly individuals [4]. Here, we aim to highlight how certain hypoxia interventions can improve cardiovascular functions and thus may represent treatment therapies for cardiovascular diseases in the aging population. For this purpose, it is paramount to examine in more detail, which characteristics of hypoxia promote or compromise health. This is of particular importance since inconsistent terminology is used in literature.

### 1.1 The different facets of hypoxia exposure

Systemic hypoxia results from low oxygen partial pressure (PO_2_) in ambient air and consequently in inspired oxygen (P_i_O_2_). Mammalian organisms can adapt to hypoxic exposures with widely varying tolerance, even among humans [10]. Depending on the hypoxic dose [6, 7, 11], hypoxia can have detrimental or beneficial effects. In humans, a commonly experienced systemic exposure to hypoxia arises during high altitude sojourns. The hypobaric hypoxia conditions of high altitudes can lead to different forms of mountain sicknesses [3, 12-14].

On the other hand, living or training in high altitude or artificial hypoxia are commonly used to improve athlete performance [15,16]. Intermittent hypoxia exposure is also applied to increase human tolerance to high altitude [2, 16-20] and may even have therapeutic value in numerous diseases [4, 18, 21].

The answer to the question, how hypoxia can induce these hugely divergent effects, is simple and complex at the same time; it is a matter of dose [6, 7, 11]. The dose of hypoxia exposure consists of several factors, including the severity of the hypoxia, the duration, and the number or frequency of exposures. The dose is furthermore crucially influenced by the resulting cumulative duration of hypoxia and additional parameters, such as arterial levels of carbon dioxide [7]. Furthermore, the mode of hypoxia administration (e.g., normobaric versus hypobaric [22]), the individual capacity of adaptation [23], the level of physical activity performed, and diet [24] as well as cross-effects with other environmental factors, such as temperature, [25] influence the outcome of exposure to hypoxia (Fig. 1). This is especially relevant when adaptations to hypoxia are studied at high altitude.

#### 1.1.1 Acute versus prolonged and chronic hypoxia

Acute exposure to hypoxia induces chemoreceptor-mediated activation of the sympathetic nervous system, resulting in increased heart rate (HR), cardiac output, peripheral resistance, and systemic arterial blood pressure [16, 26, 27]. It furthermore induces changes in the pulmonary system with increased ventilation upon a drop of blood oxygen levels detected by the carotid body [28], and pulmonary vasoconstriction which initially serves to optimize ventilation-perfusion matching, in focal hypoxia, in order to facilitate pulmonary gas exchanges [29]. Responses to hypoxia ideally lead to a reduction of oxygen-dependent processes, improved oxygen provision and protection from hypoxia-related cellular and tissue damage [2, 16]. There is currently no consensus about when the acute phase of hypoxia responses ends, since the time-course of respiratory, cardiovascular, and hematological responses differ [14]. Here, we classify exposures to hypoxia of less than 24 hours as “acute” and exposures that last longer (several days or weeks) as “prolonged”. Prolonged exposure of several days or weeks will result in sustainable hematological and vascular adaptations comprising enhanced numbers of erythrocytes and hemoglobin, as well as increased vascularization [30] and tolerance to mountain sickness builds up. However, it also results in decreased stroke volume as well as increases in heart rate, pulmonary arterial pressure, and systemic blood pressure [14]. "Chronic” hypoxia usually means long-term exposure, such as occurs in populations living in high altitudes.

#### 1.1.2 Intermittent versus continuous hypoxia

**Figure 2.**
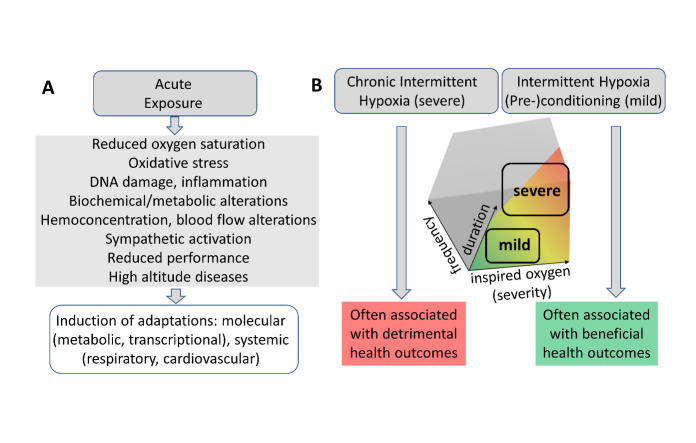
**Different doses of hypoxia elicit differential effects**. The dose thereby is a function of inspired oxygen, frequency, and duration of exposure. Acute exposure to hypoxia triggers molecular and systemic changes that can result in either beneficial adaptations or maladaptation (**A**). Accordingly, depending on the hypoxic dose (and individual vulnerabilities), intermittent hypoxia can have health-deteriorating or -promoting consequences (**B**).

Intermittent hypoxia denotes discrete periods of exposure to hypoxia, interspersed with periods of normoxia [31] or hyperoxia [16, 32]. As depicted in Fig. 2, depending on the dose, intermittent hypoxia can exert effects of completely different qualities. In contrast, continuous hypoxia refers to uninterrupted exposure to hypoxia.

#### 1.1.3 Intermittent hypoxia conditioning versus chronic intermittent hypoxia

In experimental settings, the main defining parameters are the hypoxia severity (fraction of inspired oxygen, F_i_O_2_), frequency of exposure (cycles), and duration of the cycles. Navarette-Opazo & Mitchell [7] segregated two main categories of intermittent hypoxia, severe (2-8% of inspired oxygen and between 48 and 2400 cycles per day) and moderate (more than 9% of inspired oxygen and less than 15 cycles per day).

While severe intermittent hypoxia is associated with various forms of pathology (e.g., obstructive sleep apnea syndrome or chronic obstructive broncho pneumopathy), moderate intermittent hypoxia may benefit neuronal functions, cardiovascular parameters, exercise tolerance and immune function [4, 7, 16, 33], factors thought to contribute to the overall beneficial effects of hypoxia applications [26].

We will refer to the severe, usually detrimental form of intermittent hypoxia as chronic intermittent hypoxia (CIH), and to the moderate, usually beneficial form as intermittent hypoxia conditioning. Improvement of specifically cardiovascular functions by mild intermittent hypoxia has been repeatedly demonstrated [26, 34-36], including hypertension [7, 27]. Adaptation of the cardiovascular system is a central component of the health effects of mild hypoxia also for other diseases, such as age-related neurological disorders; e.g., dementias [18].

Intermittent hypoxic-hyperoxic conditioning (IHHC) is an emerging variation of therapeutic intermittent hypoxia [16]. Hypoxic episodes in IHHC are interspersed with moderate hyperoxia, which has been hypothesized to further improve antioxidant defense induction [37] and recovery from hypoxia [38]. IHHC has been shown to be beneficial for patients with coronary artery disease [39], metabolic syndrome [40], and at least not harmful for at-risk cardiac patients [34].

Intermittent hypoxia protocols for therapeutic, preventive, performance-enhancing or acclimatization-promoting purposes have previously also been termed hypoxia/hypoxic conditioning or training. “Training” and “conditioning” refers to the application of hypoxic stress with the goal of inducing physiological adaptations to improve cellular and/or systemic resilience. Unfortunately, the terminology used for hypoxia-based interventions has been used inconsistently. This impedes systematic analysis of the literature but also public and clinical acceptance of such approaches. Harmonization of terms and protocols, and systematic testing of optimal conditions remain consistent challenges in the field.

## 2. Altitude exposure and aging

Adaptations to hypoxia are importantly mediated by reactive oxygen species (ROS) resulting in improved oxygen supply and bioenergetics by regulating hypoxia responsive elements [41]. Hypoxia conditioning can exert beneficial effects on inflammation, oxidative stress, mitochondrial functions and cell survival [16]. Inflammation [42], oxidative stress [43] and mitochondrial dysfunction [44] are all increased with age and have been discussed as main promotors of aging.

While HIFs (hypoxia-inducible factors) are essential regulators of beneficial adaptations to hypoxia, they can also contribute to maladaptation, aging, and the pathogenesis of numerous cardiovascular and cardiorespiratory diseases [45]. The involvement of HIF signaling in hypertension, atherosclerosis, aortic aneurysms, pulmonary arterial hypertension, and heart failure has been reviewed recently [46]. Accordingly, modulators of HIF signaling have potential for pharmacological intervention strategies in cardiovascular diseases [46, 47].

### 2.1 Hypoxia exposure may have beneficial effects on cardiovascular longevity and mortality

The life-span of cells [48] and the nematode *Caenorhabditis elegans* [49], can be extended in chronic hypoxic conditions.

Interestingly, the HIF system seems to be downregulated during aging in human fibroblasts [50] and in mice, indicating a potential causality between HIF signaling and aging also in mammals [51].

In humans, epidemiological studies provide some indications of possible modulation of aging by hypoxia. High altitude residence exerts various effects on mortality and a range of morbidities, such as cardiovascular diseases. Importantly, moderate and high-altitude residents are not only exposed to continuous hypoxic conditions, but they also are affected by a complex array of other physical and lifestyle factors. These potentially modulate cardiovascular function and aging, and include ultraviolet radiation and other climatic parameters, living conditions and physical activity, access to commodities and health care, etc. [52, 53].

Although clear causal links remain to be established, a growing body of evidence reports reduced mortality from cardiovascular diseases in people living at moderate altitudes. The mortality of arteriosclerotic heart disease for example has been reported to become smaller with increasing altitude of residence in New Mexico (914 to over 2135 m) for men (but not for women) [54]. Ezzati and colleagues [55] reported reduced mortality from cardiovascular diseases (ischemic heart disease) in US counties at higher altitudes, while mortality from chronic obstructive pulmonary disease increased [55, 56]. Stroke-related mortality was reduced at altitude with the strongest epidemiological repercussion between 2000 and 3500 m [57]. Reduced all-cause mortality in residents of moderate altitudes (up to around 2000 m) was primarily due to lower mortality from cardiovascular diseases in the Alpine countries Switzerland and Austria [58, 59].

Whether the main factor of beneficial high-altitude effects is chronic hypoxia, remains unknown. More information on hypoxia effects, and how hypoxia exposure can be optimized to achieve cardiovascular benefits, can be derived from interventional studies. How acute or prolonged, continuous or intermittent hypoxia, may be exploited to improve the health of the cardiovascular system, is the focus of the present narrative review.

## 3. Intermittent hypoxia conditioning - Targeting the aging cardiovascular system

Hypoxia-induced hypoxemia, sympathetic vasoconstrictor activation, pulmonary hypertension, and arrhythmias are potential health risks especially for vulnerable populations and older individuals [60]. In addition, hypoxia-induced oxidative and DNA damage [61] and high-altitude illnesses [3] are dangers of altitude/hypoxia exposure. Severe cellular hypoxia further can lead to cellular dysfunction and cell death, mechanisms central in the pathogenesis of cardiovascular diseases such as atherosclerosis, pulmonary arterial hypertension, or heart failure [62].

On the other hand, particularly the cardio-protective effects of intermittent hypoxia conditioning [4, 63, 64] may contribute to its potential to increase aerobic capacity and exercise tolerance [65], as well as to improve a range of pathologies [66-72].

In the following sections we aim to summarize the cardiovascular benefits and potential risks of intermittent hypoxia for older people.

### 3.1 Methods

Literature searches were performed using Pubmed until 31^st^ December 2022. The following search terms were included: (altitude OR “continuous hypoxia” OR “hypoxic preconditioning” OR “intermittent hypoxia” OR “hypoxic conditioning” OR “hypoxia preconditioning” OR “hypoxia conditioning” AND cardiovascular OR heart OR vascular OR hypertension OR “blood pressure” OR “arterial pressure” OR vascular* OR cardiac OR coronary OR stroke AND aging OR elderly OR older OR senior).

Pre-selection of original work that according to the title or abstract contained information on intervention studies involving continuous or intermittent hypoxia or high-altitude exposure yielded 221 results. Moreover, some papers were selected from the reference list of chosen manuscripts. In total, 53 studies were finally selected from this list after application of all inclusion and exclusion criteria (see below). The literature research was concluded on 31.12.2022 and publications until this time were included.

Initial results were screened for relevance, and duplications were eliminated. To identify studies associated with exposure to hypoxia and aging or performed in older people, the following approaches were applied:

Only intervention studies involving hypoxic conditions in humans and with a focus on cardiovascular outcomes were considered. A clear focus on aging (e.g., correlations with age, young versus older group comparison) or inclusion of age groups with an average age of at least 50 years were further requirements for inclusion. A minimum sample of 8 participants in the aged group and a minimum hypoxic severity corresponding to an altitude of 1500 m or F_i_O_2_ of 17.5% were required. Since the hypo- vs normo- baric difference occurred mainly for oxygenation and ventilatory variables rather than cardiovascular ones [73], studies using both hypobaric and normobaric hypoxic interventions were included.

### 3.2 Cardiovascular response to acute exposure *to hypoxia*

Most studies on hypoxia interventions on older individuals with reported cardiovascular outcomes were assessed during an acute hypoxia exposure lasting ≤ 24 h (Table 1). The most studied cardiovascular parameters were HR, blood pressure, and pulmonary arterial pressure (PAP).

Hypoxia-induced pulmonary vasoconstriction (HPV) is a well-described response of pulmonary vasculature to a decreased oxygen availability. This response optimizes ventilation-perfusion matching by redirecting blood perfusion towards non hypoxic alveoli during local hypoxia [29, 74]. However, during altitude exposure or systemic hypoxia, a generalized HPV leads to increases in PAP and pulmonary vascular resistance (PVR) [29, 74]. Older individuals have been reported to respond to hypoxia with larger PAP and PVR increases than their younger counterparts [75, 76]. This is probably due to several factors, such as more pronounced pulmonary vascular remodeling [77], hypoxia-induced hypoxemia [78], and/or increased oxygen sensing through higher ROS production [75] in older people. However, HPV was not always observed in older individuals. Indeed, the study reporting the shortest exposure (10 minutes) showed no change in pulmonary hemodynamics after exposure to F_i_O_2_ = 16% [79]. This may be explained by a smaller hypoxic stimulus and the biphasic HPV response, with an immediate increase (of limited magnitude) followed by a slower but progressively larger increase, after 30-60 min [80].

The hypoxic cardiac response (HCR) is defined as the ratio between HR and arterial oxygen saturation and may be lower in older than younger individuals [23, 81-83]. However, one study reported no difference in HCR between 25- and 65-years old individuals [84]. A reduced or blunted HCR could explain the lack of changes in HR when older people ascended from 898 m to 2632 m in cable car [85].

HR increases in hypoxia; and this is also true for older healthy individuals, older patients with heart diseases and older smokers during 4-5 h flight simulation [86]. Similarly, HR increased at 1980 m in trekkers mainly aged above 50 years (70%) after a walking ascent from 1400 m [87]. However, similar cardiac hemodynamics responses during maximal exercise testing have been reported in older individuals at 2750 m compared to 1400 m [88]. This reduced cardiac responses, when compared to those in younger subjects, may ultimately lead to a larger decrease in exercise capacity [88]. The discrepancy between studies may be explained by the time and modality of exposure, since increased HR in older individuals was reported only after prolonged hypoxia exposure and/or when combined with physical activity [87]. Although hypoxia is known to alter the cardiac autonomic control via sympathetic arousal or a reduction in parasympathetic drive [86], aging reduces intrinsic HR and β-adrenergic responsiveness [89] and hence limits HCR. Interestingly, less arrhythmic events were reported in older than younger individuals during car descent from 5050 m to 2950 m suggesting that the aging-associated blunted HCR could beneficially modulate cardiac rhythm [90].

Regarding the systemic circulation, hypoxia induces a biphasic systemic blood pressure response, mediated by two antagonistic mechanisms. On one hand, there are the pressor mechanisms, mainly due to sympathetic activation triggered by hypoxia-induced chemoreflex stimulation [91]. These mechanisms may be modulated by endothelial dysfunction due to hypoxia-induced oxidative stress [92] or hemorheological changes [9]. These mechanisms could be altered by commonly prescribed antiaggregant and adrenergic blockers in older individuals. Antiplatelet drugs, which are commonly prescribed to older individuals, may (beside the antithrombotic effect) attenuate endothelial dysfunction, inflammation of the vascular wall, and progression of atherosclerosis [93, 94]. Medical consultation regarding relevant medication is required before hypoxia exposure. On the other hand, a - potentially sex- and age-dependent - compensatory vasodilation due to hypoxia-induced nitric oxide (NO) release is triggered [95]. Aging decreases compensatory vasodilation, and in a larger extent in older women than in men [96, 97]. Moreover, blunted peripheral vasodilation during graded systemic hypoxia was demonstrated in a group of 10 (4 women) older individuals [98]. Compensatory vasodilation seems to be more efficient in younger women than men, but this difference seems to be lost at older age [96, 97]. It has further been suggested that certain pathologies, such as coronary artery disease could blunt exercise-induced hyperemia in hypoxia [99]. However, in this later study, young healthy controls (mean age = 23 years old) were compared to older patients (mean age = 56 years old), making it difficult to separate disease from age effects. As a direct consequence of the hypoxia-mediated vasodilator and pressor mechanisms, blood pressure is expected to change during and after hypoxia interventions, depending on the duration and severity of the exposure. A reduced blood pressure was reported in older (over 60 years) trekkers only, but not in younger ones, at 1980 m after a 580 m walking ascent [87] and in older people during acute exposure to 2500 m [100]. Conversely, at higher altitude, no blood pressure change was observed at 2632 m compared to 898 m in older individuals [85]. During longer exposure, pressor mechanisms generally overcome vasodilatory responses, explaining a reported increased blood pressure after 24 h exposure [101]. In this study, older participants only showed an elevation in systolic blood pressure while younger participants exhibited an elevation of both systolic blood pressure and diastolic blood pressure [101]. Overall, the decreased or stable blood pressure only in the older group suggests that older individuals may be less sensitive to hypoxia-mediated pressor mechanisms, despite their reduced compensatory vasodilation.

**Table 1:** Effects of acute hypoxic exposure on the aging cardiovascular system.

Subjects (if not specified, men and women were included)	Age (number), design	Hypoxia exposure	Main results	Ref.
Healthy moderate altitude dwellers (1400 m)	66 (12) Cross over design	Maximal incremental exercise at 1400 m and 2750 m with and without 50 mg Sildenafil. Hypobaric hypoxia	Decreased exercise capacity at higher altitude without changes in cardiac hemodynamic. Sildenafil only improves right ventricular function.	[88]
Healthy individuals	Young men: 27 (10) Young women: 25 (12) Older women: 63 (10) Group comparison	5 min at 10% O_2. _Normobaric hypoxia	Blunted hypoxia-related increase in peripheral blood flow in older women.	[97]
Healthy individuals, patients with stable heart diseases and smokers	Healthy non-smokers: 67 (13) Patients: 62 (9) Smokers: 61 (12) Cross over design	4-5 h flight simulation (corresponding to 2133 m) or placebo intervention. Hypobaric hypoxia	Higher HR and lower RMSSD during hypoxic exposure.	[86]
Healthy lowlanders	Older: 69 (8) Young: 25 (8) Group comparison	5 min with an alveolar oxygen pressure held at 40 mmHg. Normobaric hypoxia	Reduced HCR in older individuals.	[81]
Healthy males, shiftwork (7 days HA/7 days SL)	Older: 48 (15) Young: 30 (18) Group comparison	Ascent from 2950 to 5050 m in 41 min and descent in 39 min. Hypobaric hypoxia	Less arrhythmic events in older group during descent.	[90]
Healthy non-acclimatized men	50-64 (20) Longitudinal study	Car ascent from 898 m to 2632 m. Hypobaric hypoxia	No change at 2632 m compared to 898 m for SBP, DBP, and HR.	[85]
Trekkers (males and females)	70% of trekkers were over 50 years old (130). Age comparison	Ascent in cable car from 650 m to 1400 m and by walking from 1400 m to 1980 m. Hypobaric hypoxia	At 1980 m, SBP decreased compared to 650 m only in participants aged > 60, while DBP decreased only in trekkers aged >70. HR increased in all age categories.	[87]
Healthy normotensive	Children: 9 (8) Young: 40 (9) Older: 65 (10) Group comparison	24 h exposure at 2950 m, continuous measurements. Hypobaric hypoxia	Older participants showed an increase in SBP at altitude while other groups had an increase in SBP and DBP.	[101]
Healthy men	Young: 20 (12) Older: 56 (9) Group comparison	20 min isocapnic hypoxia (P_ET,O2 _= 50 mmHg)	Higher PAP in older group.	[75]
Healthy individuals	Young: 21 (8) Older: 65 (8) Group comparison	2 h flight simulation (2438 m). Hypobaric hypoxia	Higher systolic PAP in older group.	[76]
Healthy individuals	Young: 25 (16) Older: 65 (15) Group comparison	Maximal incremental exercise test in normoxia and hypoxia (12% O_2_). Normobaric hypoxia	Similar cardiac (and ventilatory) responses to hypoxia in young and older participants.	[84]
Healthy individuals	Premenopausal women (1152) and age-matched men <50 years (1821) Menopausal women (734) and age-matched men >50 years (1068) Cross sectional study	4 min resting hypoxia (11.5% O_2_) followed by 4 min of submaximal exercise (30% VO_2max_) in hypoxia (11.5% O_2_). Normobaric hypoxia	Decreased HCR at rest and at exercise (HVR increased at rest and exercise in men).	[23, 82]
Healthy individuals	First evaluation: 44 (30) Second evaluation: 54 (30) Longitudinal study	4 min resting hypoxia (11.5% O_2_) followed by 4 min of submaximal exercise (30% VO_2max_) in hypoxia (11.5% O_2_). Normobaric hypoxia	Decreased HCR and increased HVR only at exercise with aging.	[23, 82]
Healthy lowlanders	HAPE susceptible (HAPE+): 53 (19) Control: 46 (18) Group comparison	2 h rest and bicycle ergometer test in supine position: 25βW increased by 25βW every 2βmin to exhaustion with 12% O_2. _Normobaric hypoxia	Stronger systolic PAP increase in HAPE+ at rest and during exercise.	[102]
Healthy individuals	HAPE+: 51 (11) Control: 53 (20) Group comparison	30 min breathing hypoxic gas (SpO_2_ = 80-75%, similar to 4000 m). Normobaric hypoxia	Systolic PAP increased and right myocardial performance decreased in HAPE+.	[103]
Healthy individuals	Young: 24 (12) Older: 63 (10) Group comparison	3 x 15 min isocapnic hypoxia (SpO_2_ to 90, 85 or 80%). Normobaric hypoxia	Lack of peripheral vasodilation during graded systemic hypoxia with aging not mediated by the sympatho-adrenal system.	[98]
General population (women only)	Premenopausal with, 32 (169), and without, 36 (336) oral contraception, Postmenopausal with, 58 (69), and without, 61 (428) Group comparison	4 min resting hypoxia (11.5% O_2_) followed by 4 min of submaximal exercise (30% VO_2max_) in hypoxia (11.5% O_2_). Normobaric hypoxia	Reduced HCR in both older groups.	[83]
**Patients group**				
Pulmonary arterial or chronic thrombo-embolic pulmonary hyper-tension patients	Patients: 62 (25) Healthy: 60 (16) Group comparison	16% O_2_ (2600 m) for 10 min. Normobaric hypoxia	Stronger PaO_2_ reduction in patients in hypoxia. No difference in pulmonary vascular resistance, mean PAP and cardiac output.	[79]
Coronary artery disease patients	Patients: 56 (8) Healthy: 23 (10) Group comparison	Patients: Exercise at 16.5% O_2_ Healthy: Exercise at 12.5% O_2. _Normobaric hypoxia	Exercise induced hyperemia increased in healthy participants at altitude but not in patients.	[99]
Coronary artery disease patients	Patients: 51 (23) Healthy: 53 (23) Group comparison	Maximal symptom-limited bicycle stress test at 1000 and 2500 m. Hypobaric hypoxia	Maximal HR and BP were similar between both groups.	[104]
Grade 1 hypertensive patients	52 (89) Cross over design	6 min walking test in altitude after 1 day at 3260 m. Hypobaric hypoxia	BP at exercise increased in altitude, angiotensin receptor blocker-calcium channel blocker prevented this effect without decreasing exercise performance.	[105]
COPD patients	Young: 30 (12) Older: 68 (14) COPD: 67 (12) Group comparison	5 min isocapnic hypoxia (P_ETO2_ = 50 mmHg). Normobaric hypoxia	Reduced cerebrovascular reactivity to hypoxia in older and COPD individuals. No hypoxia-related change in BP in older or COPD.	[106]
Volunteers, 85% with known coronary artery disease or at high risk	68 (20) Longitudinal study	Test in acute simulated altitude: 2500 m Normobaric hypoxia Followed by 5 days at 2500 m Hypobaric hypoxia	Increased resting HR in simulated altitude and after 5 days of exposure. Decreased resting SBP and DBP in acute altitude. Increased PAP and sympathetic activation acutely and chronically.	[100]

HR: heart rate, RMSSD: root mean squared successive difference, BP: blood pressure, SBP: systolic blood pressure, DBP: diastolic blood pressure, PAP: pulmonary arterial pressure, HCR: hypoxic cardiac response, HVR: hypoxic ventilatory response, HAPE: high-altitude pulmonary edema, MVV: maximal voluntary ventilation, COPD: chronic obstructive pulmonary disease, PaO_2_: arterial oxygen pressure, and P_ETO2_: end tidal oxygen pressure.

### 3.3 Cardiovascular response to prolonged exposure to hypoxia

Longer than 24 h exposure to hypoxia is associated with maintenance or change of several cardiovascular responses (Table 2). For example, persistent increases of PAP or PVR due to HPV have been shown even after 11 days of trekking to Mount Kilimanjaro (5893 m) [107, 108], 2 weeks acclimatization between 1800 and 4200 m [109], or 5 days at 2500 m [100]. Conversely, PAP was not increased after 3 weeks at 1700 m in older metabolic syndrome patients [110], suggesting that this altitude was not sufficient to trigger HPV and is safe regarding pulmonary vascular function. The impact of age on this later response is less clear: Stewart et al. (2020) reported higher PVR and PAP in older individuals compared to younger at sea level and altitude [108] while Coffman et al. (2019) did not report any age difference for the same altitude exposure [107]. The HPV did not appear to exceed the limits of the right ventricular compensatory reserve in both young or old healthy trekkers during an 11-day trek at high altitude [108]. This is consistent with previous reports in young individuals [111, 112]. Regarding clinical populations, one study demonstrated similar right ventricular dilation in coronary arteries which was associated with an increased PAP, in both patients and healthy individuals, suggesting that HPV is not exacerbated in coronary artery old patients [109].

In line with the above-described reduced HCR in older individuals, young subjects had larger increases in HR and cardiac output, when compared to their older counterparts, during an 11-day trek up to 5893 m [108], supporting a lower cardiac sympathetic response in older individuals and/or intrinsic myocardial changes with aging. However, although reduced in older individuals, increases in HR and sympathetic activation are still apparent during prolonged exposure (e.g., 5 days at 2500 m) [100].

Based on the biphasic blood pressure response, an increased blood pressure is expected during prolonged hypoxia exposure. This was experimentally confirmed in older people by several studies at various altitudes (2000 to 3260 m) and with various durations (2 to 8 days) [100, 113-116]. Such elevated blood pressure is accompanied by a decrease in baroreflex sensitivity [115]. Not surprisingly, milder hypoxia affects blood pressure less, especially when altitude exposure is combined with physical activities (i.e., hiking): no adverse changes in blood pressure were observed after 1 week at 2000 m or at 1700 m [117, 118]. Unfortunately, none of these studies have compared the blood pressure responses to prolonged exposure in old and young individuals. Moreover, compared to baseline, decreased systolic blood pressure and diastolic blood pressure have been observed after 3 weeks at 1700 m in older metabolic syndrome patients [110]. This reduction in blood pressure was surprisingly accompanied by a transient decrease in flow mediated dilation [119]. In this context, one can hypothesize that an increased vasodilatory state after 3 weeks in altitude in older patient may reduce blood pressure but impair flow mediated dilation probably due to age-related decline in vascular reactivity.

**Table 2:** Effects of prolonged hypoxic exposure on the aging cardiovascular system.

Subjects (if not specified, men and women were included)	Age (number), design	Hypoxia exposure	Main results	Ref.
Healthy individuals residing at SL	Older: 59 (13), Young. 32 (14) Group comparison	11 days trek to Mt Kilimanjaro (5893m). Hypobaric hypoxia	Rapid myocardial adaptations to altitude were mostly similar between young and older trekkers. Higher PVR in older trekkers at all timepoints.	[108]
Healthy individuals residing at SL	Older: 59 (13), Young: 32 (14) Group comparison	11 days trek to Mt Kilimanjaro (5893m). Hypobaric hypoxia	Trend towards higher extravascular lung water in older group despite similar systolic PAP.	[107]
Healthy dwellers living between 600 - 900 m	High altitude group: 67 (10) Low altitude group: 64 (10) Group comparison	1 week hiking program at 600 m or 2000 m, 2.5 - 5 h increasing hiking time for 1 week. Hypobaric hypoxia	Cardiopulmonary and metabolic responses to exercise increased for 1 week hiking program in altitude. No change in SBP or DBP.	[117]
Healthy individuals	Young: 23 (7) Older: 61 (9) Group comparison	Test at SL, after 3 days at 2200 m and after 14 days climbing to 4200 and 5642 m. Hypobaric hypoxia	HVR is lower in older, blood dopamine and dihydroxyphenylalanine higher in older at SL with reduced change in altitude.	[120]
Healthy inhabitants of alpine regions	> 60 (24) Longitudinal study	One ascent of 500 m in less than 3 h per week for 9 months. Hypobaric hypoxia	No change in the whole group. Considering only participants with pathological or borderline values, SPB tended to improve.	[121]
General population	70 (97) Longitudinal study	4 days (2500 m). Hypobaric hypoxia	BP increased during the stay in altitude. No adverse cardiac signs or symptoms occurred.	[113]
**Patients group**				
Coronary artery disease patients	Patients: 53 (8) Healthy: 41 (7)	2 weeks of acclimatization between 1800 and 4200 m. Hypobaric hypoxia	Similar pulmonary and cardiovascular responses in patients and controls.	[109]
Patients with known coronary artery disease or at high risk	68 (20) Longitudinal study	Test in acute simulated altitude (2500 m), followed by 5 days at 2500 m. Hypobaric hypoxia	Increased resting HR in simulated altitude and after 5 days of continuous hypoxia. Decreased resting SBP and DBP in acute altitude. Increased PAP and sympathetic activation, acutely and chronically.	[100]
Grade 1 hypertensive patients	56 (89) Longitudinal study	3 days at 3260 m. Hypobaric hypoxia	24 h BP increase in altitude, angiotensin receptor blocker-calcium channel blocker combination is effective and safe in altitude.	[114]
Grade 1 hypertensive patients	57 (55) Longitudinal study	Exercise test in altitude after 2 days at 3260 m. Hypobaric hypoxia	Increased BP response to exercise under hypobaric hypoxia.	[122]
COPD patients	64 (37) Longitudinal study	2 days at 2590 m. Hypobaric hypoxia	Increased SBP and decreased BP variability and BRS.	[115]
OSA patients	62 (34) Longitudinal study	5-6 days at 1860 m followed by 7-8 days at 2590 m. Hypobaric hypoxia	Increased SBP and cardiac arrhythmias at altitude.	[116]
Metabolic syndrome	55 (18) Longitudinal study	3 weeks at 1700 m. Hypobaric hypoxia	Reversible decreased FMD after altitude exposure.	[119]
Metabolic syndrome	55 (18) Longitudinal study	3 weeks at 1700 m. Hypobaric hypoxia	No adverse cardiovascular effects, no increases of PAP. Decreased SBP and DBP after altitude exposure.	[110]
Metabolic syndrome	1700 m group: 55 (36) SL group: 55 (35) Group comparison	3 weeks at 1700 m or SL with 10 h hiking/week. Hypobaric hypoxia	No group difference after 3 weeks intervention in BP or HR.	[118]

HR: heart rate, BP: blood pressure, SBP: systolic blood pressure, DBP: diastolic blood pressure, PAP: pulmonary arterial pressure, HVR: hypoxic ventilatory response, BRS: baroreflex sensitivity, FMD: flow-mediated dilation, OSA: obstructive sleep apnea, COPD: chronic obstructive broncho pneumopathy, SL: sea-level.

### 3.4 Effects of intermittent hypoxia-normoxia or hypoxia-hyperoxia on the aging cardiovascular system

Among the various types of hypoxic interventions described in chapter 1.1, single session protocols of intermittent hypoxia should be differentiated from other ones. Responses may largely differ after one session of intermittent hypoxic exposure than after an entire protocol composed of several sessions. Only two studies investigated effect during and/or after one intermittent hypoxia in older individuals in association with cardiovascular outcomes [123, 124]. Liu et al. (2020) reported that the cerebrovascular response to hypoxia was diminished with age [123]. These findings confirm previous results showing a decreased cerebrovascular reactivity to hypoxia in older individuals, compared to young ones, during 5 min isocapnic hypoxic exposure [106]. Regarding blood pressure, no reduction was reported in pre-hypertensive older people after only one session of intermittent hypoxia [124]. However, in healthy older individuals, intermittent hypoxia reduced systolic blood pressure and diastolic blood pressure during the first and the last bout (5 bouts in total) of the first session [123]. This suggests that during acute exposure (5 min), hypoxia-induced peripheral vasodilation [125] is more prominent than the sympathetic-mediated vasoconstriction elicited by hypoxia [126]. Carbon dioxide seems to play a determinant role in acute blood pressure responses to hypoxia since poikilocapnic hypoxia induced peripheral vasodilation and blood pressure decrease [123, 125] while hypercapnic hypoxia greatly increased blood pressure [127] and isocapnic hypoxia induced either an increase [127] or no changes in blood pressure [106]. Hypercapnia increased blood pressure similarly in young and older subjects while hyperventilation-induced hypocapnia decreased blood pressure, but to a lower extent in older people [128]. Hence, poikilocapnic hypoxia, rather than hypercapnic hypoxia may be better suited to acutely decrease blood pressure. This could affect the therapeutic efficiency of devices such as hypoxic tents or rebreathing systems. Overall, it highlights the importance of considering other stressors associated with the modality of hypoxic exposure (e.g., chronic intermittent hypoxia in patient with obstructive sleep apnea is associated with hypercapnia and acidosis while intermittent hypoxia conditioning is associated with hypocapnia and alkalosis [129]).

Intermittent hypoxia conditioning (i.e., 3 to 5 sessions per week), including IHHC protocols, have been recently investigated as an innovative therapeutic strategy for several dysfunctions, especially of vasculature. Hence, most of the studies using these interventions focus on hematological and/or blood pressure changes (Table 3). Ten studies investigated blood pressure changes after intermittent hypoxia conditioning in older people [34, 36, 39, 40, 130-133]. Six of them reported a blood pressure decrease following intermittent interventions in older healthy people or patients with coronary artery diseases, prior myocardial infarction, or metabolic syndrome [36, 39, 40, 130, 134, 135]. Four other studies reported either a trend toward a reduction in blood pressure in healthy people or patients [34, 131] or no change [132, 133]. Dudnik et al. (2018) reported a significant decrease in diastolic blood pressure but not in systolic blood pressure (p = 0.07) in cardiac outpatients after 15 sessions of IHHC [34]. After a long period of intermittent hypoxia interventions (24 weeks), Timon et al. (2022) merely observed a trend of decreased systolic blood pressure and diastolic blood pressure (P = 0.068 and P = 0.057, respectively) [131]. This discrepancy may be explained by the 45 min-long exposure to hypoxia (F_i_O_2_ = 16%) during a continuous period rather than with the recommended intermittent pattern. Another study using long periods of intermittent hypoxia (25 min) coupled with exercise failed to improve blood pressure after 18 sessions [133]. Indeed, to reduce blood pressure, hypoxic cycles of 2 to 10 min have previously been suggested for higher safety and efficiency in older individuals [9]. Moreover, a study investigating IHHC effects in very old participants (mean age 81 ± 8 yr old) reported enhanced exercise endurance in the absence of blood pressure changes [132]. This may be due to an insufficient vascular reserve in very old individual or patients (e.g., diabetes) to benefit from hypoxic stimuli. Indeed, an attenuated hyperemic vasodilatory response due to blunted NO signaling in hypoxia was reported with aging [136]. Underlying mechanisms suspected to regulate blood pressure mainly relate to NO availability, due to enhanced endothelium NO synthase [137], erythrocyte NO synthase [133], and sympatholysis [138]. Furthermore, evidence of reduced arterial stiffness [40] and reduced low grade inflammation [131] after IHHC may contribute to benefits on vascular function and blood pressure.

Hematological and hemorheological changes are also suspected to be involved in blood pressure changes after and during hypoxia exposure [9]. Increased blood viscosity, which depends largely on hematocrit (Hct) and red blood cell (RBC) behavior [139], leads to a rise in wall shear stress, which increased NO [140]. Increases in RBC count, Hct, and hemoglobin concentration (Hb) have been reported after only five days of intermittent hypoxia conditioning, while continuous exposure (same total hypoxic exposure time of 350 - 360 min) did not induce any change [141]. These are supported by other studies; e.g., Burtscher et al. [36] reported an increase in RBC count and Hb without changes in Hct and Glazachev et al. [39] demonstrated higher relative reticulocyte amounts after IHHC compared to the control intervention, suggesting stimulated erythropoiesis. However, one study did not report any change following 10 sessions of isocapnic intermittent hypoxia [130]. Further studies and comparable protocols, techniques and controls will be crucial to fully characterize hematological and hemorheological changes following intermittent hypoxia.

Sympatholysis is another underlying mechanism potentially responsible for hypoxia-induced blood pressure changes. This is supported by changes in HRV. The root mean squared successive difference, a reflect of vagal outflow, increased after 4 weeks of intermittent hypoxia conditioning (+71.6 ± 52.5%), while the changes 4- and 8-weeks post intervention are less clear [134].

Despite inconsistent results on the efficiency, partially owed to differences in study-populations and protocol settings, well-calibrated intermittent hypoxia conditioning interventions are generally considered safe in older individuals (Table 3 references) [141]. An efficient strategy to reduce blood pressure in older individuals (but not too old [132]), seems to be the repeated application of short hypoxic cycles (2-10 min, F_i_O_2_ ≥ 10%) over several weeks.

**Table 3:** Effects of intermittent hypoxia-normoxia or hypoxia-hyperoxia on the aging cardiovascular system.

Subjects (if not specified, men and women were included)	Age (number), design	Hypoxia exposure	Main results	Ref.
**Single session**				
Healthy and unacclimatized to altitude	Older: 71 (12) Young: 24 (13) Group comparison	5 times (5 min 10% O_2 _- 5 min 21% O_2_). Normobaric hypoxia	Hypoxia-responsive cerebrovascular reserves were diminished at older age. Acute hypoxic exposure induced a modest decrease in BP.	[123]
Prehypertensive but otherwise healthy older individuals	69 (14) Cross over design	6 times (5 min 10.5 % targeting 70-80% SpO_2_ - 5 min in hyperpnoea at 60% MVV with partial rebreathing). Normobaric hypoxia	SBP tended to be lower (p=0.09) after one acute session of IH-hyperpnoea than one placebo session.	[124]
**Multiple sessions**				
Healthy, untrained and unacclimatized to altitude	67 (15) Cross over design	5 days with a total time exposure of 350-360 min at SpO_2_ of 85% with either IH (normoxic phase: 95% SpO_2_) or CH. Normobaric hypoxia	CH and IH did not induce cardiac damage in older individuals. RBC count, Hct and [Hb] increase only after IH in older individuals.	[141]
Healthy lowlanders without cardiac or pulmonary diseases	Active group: 67 (14), Sedentary group: 61 (22) Group comparison	4 times a cycle of 5 min 12% O_2 _isocapnic - 5 min 21% O_2_ for 10 days. Normobaric hypoxia	Intermittent hypoxia reduced SBP, at rest, submaximal exercise and during acute hypoxic exposure in sedentary group. Decreased DBP at submaximal exercise in the sedentary group. No hematologic changes.	[130]
Healthy lowlanders*	Intermittent hypoxia group: 70 (19) Control group: 70 (19) Group comparison	45 min sessions of 16% O_2_, 3 sessions/week for 24 weeks. Normobaric hypoxia	Reduced low-grade inflammation and non-significant decrease in SBP (p=0.068) and DBP (p=0.057) after intervention.	[131]
Healthy individuals	Intermittent hypoxia group: 56 (8) Control group: 56 (8) Group comparison	F_i_O_2_ continuously decreased from 21 to 10%. 4 sessions/week for 4 weeks: 6 x 5 min hypoxia alternated with 5 min normoxia per session. Normobaric hypoxia	Intervention increased vagal outflow. Decreased heart rate variability during hypoxia exposure.	[142]
Inactive participants	Exercise group: 56 (18) Intermittent hypoxia + exercise group: 56 (16) Group comparison	Exercise: 1h, 3 days/wk for 10 weeks. Intermittent hypoxia: 6 times a cycle of 5 min with a F_i_O_2_ targeting 90-80% SpO_2_, 2-3 days/wk for 10 weeks. Normobaric hypoxia	Addition of IHC resulted in more favorable changes in SBP, cardiorespiratory fitness and lipid profile. Unclear changes in resting parasympathetic activity and pulse wave velocity.	[134]
**Patients group**				
Normally physically active male lowlanders with or without prior myocardial infarction	Intermittent hypoxia group: 59 (8), Control group: 61 (8) Group comparison	5 sessions (3-5 min at 10-14% O_2_ alternated with 3 min 21% O_2_) /week for 3 weeks. Normobaric hypoxia	Reduced HR and SBP during submaximal exercise in intermittent hypoxia group only. Increased RBC count and Hb concentration in intermittent hypoxia group.	[36]
46 patients with coronary artery diseases	IHHC group: 64 (27) Control group: 63 (19) Group comparison	3 sessions/week for 5 weeks, per session 5-7 times (4-6 min 10-12% O_2 _alternated with 3 min 30-35% O_2_). Normobaric hypoxia	IHHC reduced SPB and DBP, increased % of reticulocyte and induced enhance lipidic profile.	[39]
Patients of a geriatric care unit without heart or pulmonary diseases	IHHC group: 81 (18), placebo group: 83 (16) Group comparison	Individualized multimodal training program + placebo or IHHC for 5-6 weeks, overall 14-15 sessions, per session 4-6 min 12% O_2 _alternated with 1-2 min 35% O_2_. Normobaric hypoxia	Greater increase in exercise endurance with IHHC than placebo. No significant decrease in SPB and DBP after IHHC.	[132]
Cardiology outpatients with comorbidities (hypertension, diabetes, obesity, COPD)	IHHC group: 66 (15), Control group: 65 (14) Group comparison	3 sessions/week for 5 weeks, per session 5-7 times 4-6 min 11-12% O_2 _alternated with 3 min 30-33% O_2. _Normobaric hypoxia	Trend toward a reduced SPB in the IHHC group and reduced DBP in IHHC group.	[34]
Diabetes mellitus^#^	61 (12) Longitudinal study	Hypoxic exercise: 4 sessions/week for 6 weeks; per session 3 times 25 min 15.4-12.7% O_2 _alternated with 5-10 min normoxia. Normobaric hypoxia	No change in BP after the intervention while dysregulation of NOS-activation in erythrocytes partially restored after the intervention.	[133]
Metabolic syndrome	IHHC: 57 (32) Placebo: 60 (33) Group comparison	5 sessions/week for 3 weeks¸per session 5-8 times 4-7 min 11-12% O_2 _alternated with 2-4 min 30-35% O_2. _Normobaric hypoxia	IHHC decreased SBP and DPB. Small evidence that arterial stiffness is reduced after IHHC.	[40]
Overweight*	Endurance training normoxia: 64 (13) Endurance training hypoxia: 60 (12) Group comparison	8 weeks endurance training in normoxia or hypoxia (15% O_2_). 3 training/wk 30-40 mn at 60-70% VO_2peak. _Normobaric hypoxia	No change in BP after training. Reduced SBP during constant work exercise only in hypoxic group.	[135]

HR: heart rate, BP: blood pressure, SBP: systolic blood pressure, DBP: diastolic blood pressure, IHHC: intermittent hypoxia-hyperoxia conditioning, NOS: nitric oxide synthase, RBC: red blood cell, Hct: hematocrit, IH: intermittent hypoxia, F_i_O_2_: inspired oxygen fraction, SpO_2_: oxygen pulse saturation, VO_2peak_: peak oxygen consumption, [Hb]: hemoglobin concentration. * Studies with continuous hypoxic exposure repeated several times a week. ^#^ study with uncommon long hypoxic cycle (25 min) interspersed with normoxic exposure.

### 3.5. Cardiovascular implications on exercise performance and high-altitude illnesses of older individuals

As outlined above, oxidative stress and inflammatory processes contribute importantly to aging of the cardiovascular system, and in turn, to the development of various cardiovascular diseases; e.g., atherosclerosis, systemic hypertension, myocardial infarction, and stroke [143]. Consequently, performance limitations, high-altitude illnesses and cardiovascular adverse events may be expected to occur more frequently in older high-altitude trekkers and climbers, since oxidative stress and inflammation are both considered hallmarks of aging. Changes in cardiorespiratory fitness (CRF), assessed by the maximal aerobic power (VO_2_max) of an individual, are most striking changes observed with aging [144-146]. An about 5% VO_2_max decline per decade above 30 years may be related to the aging process per se, but an about 10% decline is observed in the majority of all individuals, i.e., independent of health and training status [144, 146]. Aging-related reduction in HRmax (3 - 5% per decade) seems to play a major role for the decline in VO_2_max [147, 148], possibly accompanied by reduced inotropic and Frank-Starling reserve [149]. Whether the maximal arteriovenous oxygen difference is impaired with aging or simply represents an artifact of diminished lean body mass with aging remains to be elucidated [144, 149]. Moreover, individual CRF decreases by about 10% every 1,000 m of additional gain in altitude above 1,500 m [150] and physiological exercise responses (e.g., HR, blood pressure, and ventilation) are significantly increased at high altitude [151]. Thus, the aging related CRF decline may become particularly relevant at high altitude, likely increasing the risk of severe cardiovascular adverse events in those with pre-existing diseases and low baseline CRF when trekking or climbing in high-altitude regions [152, 153]. Whether and how aging of the cardiovascular system modulates the incidence of high-altitude illnesses remains less clear. Richalet and colleagues emphasized that aging is not contraindicated for high-altitude sojourns in absence of relevant comorbidities and if the individual CRF is compatible with the intensity of the expected physical demand of the trekker/climber [23]. Findings from a relatively large cohort indicate that a high self-reported level of exertion during the ascent to high altitude was associated with a nearly 25-fold increased risk of AMS [154]. As the self-reported exertion level is closely related to the individual’s CRF, low CRF in older mountaineers may provoke AMS development, in particular when these individuals are forced to keep pace with their younger and/or fitter peers [150, 154]. A recent systematic review found no association between older age and the risk of high-altitude illnesses, in particular with regard to the development of AMS [155]. In accordance with those findings, a pooled analysis of five prospective randomized controlled trials did not see any AMS risk increase with aging [156]. In contrast, Leshem et al. reported more frequent evacuations due to high-altitude illnesses among older trekkers in the Everest region [157]. Some support for a potentially elevated HAPE risk comes from recently reported echocardiographic assessments, i.e., systolic pulmonary pressure and peak in tricuspid regurgitation velocity [158] and the observation of elevated extravascular lung water, likely due to increased pulmonary vascular resistance in older high-altitude trekkers/climbers [107].

Taken together, older age is not a contraindication for high-altitude sojourns and there is not an elevated risk for high-altitude illnesses. However, as aging is associated with lower CRF and a greater likelihood of cardiovascular risk factors, medical pre-travel advice and appropriate fitness training are of utmost importance to safely enjoy trekking or climbing at high altitude.

## 4. Conclusion and perspectives

Cardiovascular diseases are common consequences of aging. Here we reviewed the scientific evidence and potentials of hypoxia interventions to alleviate cardiovascular complaints in older people.

**Figure 3.**
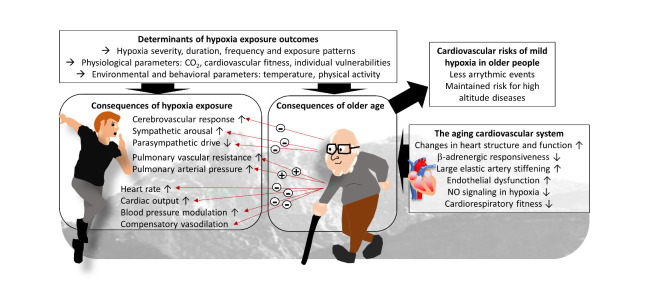
**Physiological consequences of exposure to moderate hypoxia and modulation by age**. Many factors determine physiological outcomes of hypoxia. Also age-related changes of the cardiovascular system modulate these outcomes. CO_2_: carbon dioxide, NO: nitric oxide.

Since aging is associated with various cardiovascular alterations that often increase the risk of related morbidity and mortality, it is not surprising that cardiovascular and respiratory responses in hypoxia are also altered (Fig. 3). Relevant differences include larger PAP and PVR increases in older people in hypoxia. Concurrently, HCR, HR, cardiac output, blood pressure responses and cerebrovascular reactivity seem to be frequently attenuated at higher age, partly due to age-related deficits of sympathetic modulation (β-adrenergic responsiveness) and impaired function of heart and vasculature.

However, older subjects appear to be not at higher risk for AMS. Since exercise intensity and exhaustion directly affect the prevalence and the severity of symptoms, adjustment in walking velocity (e.g., taking more time to climb a summit or for a trek) is likely protective (but has not been directly investigated) in older people who on average have a lower CRF.

Age-effects on the biphasic blood pressure response in hypoxia are complex. First, there appear to be poorly understood sex-specific differences in older people in the efficiency of compensatory vasodilation. Second, specific age-related compensatory blood pressure-reducing effects remain to be fully characterized.

During submaximal exercise - that corresponds to the intensity required for hiking or mountaineering - there is an increase in HR at a given velocity likely due to sympathetic activation. The reduced sympathetic responsiveness of older people may lead to a lower increase in absolute HR. The functional sympatholysis during exercise in hypoxia may be also blunted in older subjects. This finding suggests that older individuals may require a higher hypoxic dose during intermittent hypoxia conditioning intervention for inducing significant cardiovascular benefits (e.g., decrease in blood pressure). Safety and efficiency of increased altitude severity deserves further investigation. Therefore, the main parameter to be safely increased may be the number of sessions. Hence, age is an important factor to be considered in the development of appropriate protocols using hypoxia exposure for preventive, therapeutic or performance enhancing purposes. Importantly, besides the variation of the severity and/or duration of the hypoxic exposure, the pattern of administration is emerging as an important determinant of hypoxia outcomes. The use of repeated application of artificial hypoxia seems to maximize some outcomes as compared to continuous applications, with the repeated application of several (about 4-7) hypoxic cycles (about 2-10 min with F_i_O_2_ > 10%, interspersed with normoxic or hyperoxic episodes) per day applied across about 3 - 8 weeks, seeming particularly promising. Importantly, a threshold of physiological capacity to respond to hypoxia may be necessary to reap cardiovascular benefits from hypoxia interventions, since very old people have been reported to be relatively unresponsive to blood-pressure effects even of an IHHC intervention [132]; an important question here is, whether hypoxia-responses can be trained, i.e., improved, at higher age with adequate strategies. Other parameters such as that modulates cardiovascular outcomes, such as carbon dioxide level, low CRF, and the time of the day (which may compromise sleep quality) should be considered.

In conclusion, hypoxia-based therapies offer great potential as novel strategies to prevent and treat age-related cardiovascular diseases. Currently, however, the mechanistic understanding of such strategies is still limited, and the scientific evidence fragmented, since so many parameters modulate the outcome of hypoxia exposure (hypoxic dose, administration patterns, carbon dioxide, other environmental conditions, behavior, and vulnerability of the subjects) and physiological responses change non-linearly from the first hypoxia exposure to the end of the exposure and after. Some of the reported apparently conflicting results therefore likely are due to differences in the exposure protocols or target population-specific outcomes.

To move the field further, an essential step will be the provision of generally accepted guidelines, helping to characterize, select, adequately describe, and report hypoxia-based intervention protocols for specific study populations and designs. This will allow the conduction of comparable studies and the more systematic comparison of age-related (and other characteristics of the study population) physiological outcomes of different well-defined hypoxia-related protocols.
